# Clinical features of type A aortic dissection initially presenting with neurological symptoms: a single-center case series

**DOI:** 10.3389/fneur.2026.1802180

**Published:** 2026-06-11

**Authors:** Haoyu Zou, Yujing Zhu, Xinyu Li, Guang Zhang

**Affiliations:** Department of Neurosurgery, The First Affiliated Hospital of Harbin Medical University, Harbin, Heilongjiang, China

**Keywords:** acute stroke, aortic dissection, D-dimer, limb weakness, type A aortic dissection

## Abstract

**Background:**

Aortic dissection, a life-threatening condition, may present with neurological symptoms mimicking acute stroke, leading to delayed diagnosis and inappropriate management.

**Objective:**

To describe the clinical characteristics and outcomes of patients with type A aortic dissection (TAAD) who initially presented with neurological symptoms and were first admitted to the neurology department.

**Methods:**

We retrospectively reviewed the demographic, clinical, laboratory, imaging, treatment and outcome data of 23 patients with TAAD who initially presented with neurological symptoms and were first admitted to our neurology department between October 2019 and August 2025 and were subsequently confirmed to have TAAD.

**Results:**

The mean age of patients was 62.4 ± 12.2 years, and eight (34.8%) were women. The most common presenting symptoms were altered consciousness (15 cases, 65.2%) and limb weakness (13 cases, 56.5%), including 11 cases of left-sided weakness; only five patients (21.7%) reported chest or back pain. The median D-dimer level was 22.73 mg/L (interquartile range [IQR], 9.13–61.17). Nine patients (39.1%) received intravenous thrombolysis before the diagnosis of acute aortic dissection, and four (17.4%) underwent surgical repair. Fifteen patients (65.2%) died during hospitalization, and eight (34.8%) were discharged alive. Discharged patients were followed up for a median of 39 months (IQR, 14–57.75), during which two surgically treated patients remained alive, while four of the six non-surgically treated patients died. The overall long-term survival rate was 17.4% (4/23).

**Conclusion:**

Acute TAAD may mimic acute stroke, leading to misdiagnosis and inappropriate thrombolytic therapy. In patients without typical chest pain, atypical features such as altered consciousness, left-sided weakness, elevated D-dimer levels, and an inter-arm blood pressure difference, when present, may raise suspicion for TAAD.

## 1 Introduction

Although acute aortic dissection is relatively rare, with an annual incidence of approximately 2.78 cases per 100,000 population in China (1), it is a rapidly progressing and life-threatening condition. In particular, type A aortic dissection (TAAD) poses a high risk. If misdiagnosed or untreated, the mortality increases by approximately 1–2% for every hour of delay (2), with an overall in-hospital mortality rate of approximately 20.3% (3). Previous studies have reported that 3–6% of patients with TAAD initially present with neurological symptoms (4, 5). Such patients often lack the typical presentation of chest pain and may initially present with neurological symptoms, such as altered consciousness and limb weakness. They are therefore frequently admitted to neurology departments for presumed neurological disorders, potentially leading to delayed diagnosis and poor outcomes (6). Although current domestic and international guidelines acknowledge the possibility of neurological manifestations in aortic dissection, evidence-based strategies for early identification by neurologists remain limited. Therefore, this study aimed to summarize the clinical characteristics of patients with TAAD who first present with neurological symptoms, which is essential for improving early recognition, reducing misdiagnosis, and enhancing prognosis.

## 2 Materials and methods

### 2.1 Study population

This retrospective single-center study included patients with confirmed TAAD who initially presented with neurological symptoms at The First Affiliated Hospital of Harbin Medical University (a tertiary hospital with 4,200 beds) between October 2019 and August 2025. All patients with abnormal neurological symptoms or symptoms of stroke were admitted into Department of Neurology after an exclusion of intracranial hemorrhage on non-contrast CT and received intravenous thrombolysis if there were no contraindications. Patients with unstable vital signs accompanied by neurological manifestations, such as altered consciousness, would be admitted to the neurological ward and included in this study.

The inclusion criteria were as follows: (a) initial manifestations with neurological symptoms (limb weakness or altered consciousness) and first evaluation by a neurologist and (b) diagnosis of TAAD confirmed by post-admission imaging.

The exclusion criteria were as follows: (a) incomplete clinical data; (b) prior diagnosis of aortic dissection; and (c) pre-existing severe neurological deficit (modified Rankin Scale score ≥3 before onset).

The study protocol was approved by the Ethics Committee of The First Affiliated Hospital of Harbin Medical University. Given the retrospective study design, the requirement for informed consent was waived.

### 2.2 Data collection

Demographic, clinical, and imaging data regarding the following variables were retrospectively collected: age, sex, body mass index (BMI), medical history (hypertension, diabetes mellitus, heart disease), clinical manifestations (chest pain, limb weakness and altered consciousness), stroke severity assessed by the National Institutes of Health Stroke Scale (NIHSS) at admission, laboratory findings (D-dimer level), lifestyle-related factors (smoking and alcohol consumption) and imaging results (echocardiography, carotid ultrasound, and thoracoabdominal computed tomography angiography [CTA]). Additionally, treatment modalities (intravenous thrombolysis and surgical repair) and survival outcomes were recorded. All data were independently reviewed by two investigators, and any discrepancies were resolved through discussion to ensure accuracy.

### 2.3 Statistical analysis

All statistical analyses were performed using IBM SPSS Statistics software, version 25.0 (IBM Corp., Armonk, NY, United States). Continuous variables with a normal distribution are presented as mean ± standard deviation (SD), whereas non-normally distributed variables are expressed as median and interquartile range (IQR). Categorical variables are summarized as frequencies and percentages. Comparisons between two groups for continuous variables were performed using the independent samples *t*-test or the Mann–Whitney U test, as appropriate. Categorical variables were compared using the chi-square test or Fisher’s exact test. A two-tailed *p* value < 0.05 was considered statistically significant.

## 3 Results

### 3.1 Baseline characteristics

A total of 29 patients with aortic dissection initially presenting with neurological symptoms were admitted in the hospital between October 2019 and August 2025. Six patients were excluded because of prior diagnosis of aortic dissection, only suspicious TAAD without CT angiography of aortic arch or type B aortic dissection. Finally, 23 patients with TAAD initially presenting with neurological symptoms were included, 8 survivors and 15 patients who died during hospitalization. The cohort comprised 15 men and 8 women, with an age range of 33 to 86 years. Table 1 summarizes the demographic characteristics, clinical manifestations, vital signs, laboratory findings, and treatment modalities of all patients. The mean age of the patients was 62.4 ± 12.2 years, and eight (34.8%) were women. The mean body mass index was 24.7 ± 3.6 kg/m^2^. Regarding medical history, hypertension was the most common comorbidity (16 cases, 69.6%), followed by diabetes mellitus and heart disease (two cases each, 8.7%). Six patients (26.1%) had a history of smoking, and five (21.7%) had a history of alcohol consumption.

**Table 1:** Baseline characteristics, clinical features, and treatments of 23 patients with type A aortic dissection initially presenting with neurological symptoms.

Variable	Total (*N* = 23)	Survivors (*N* = 8)	In-hospital non-survivors (*N* = 15)	*p* value
Demographics				
Female, *n* (%)	8 (34.8%)	2 (25.0%)	6 (40.0%)	0.657
Age, years (mean ± SD)	62.4 ± 12.2	58.1 ± 14.1	64.7 ± 10.9	0.300
BMI (kg/m^2^, mean ± SD)	24.7 ± 3.6	26.8 ± 1.9	23.6 ± 4.1	0.049
Medical history				
Hypertension, *n* (%)	16 (69.6%)	7 (87.5%)	9 (60.0%)	0.345
Diabetes mellitus, *n* (%)	2 (8.7%)	2 (25.0%)	0	0.111
Heart disease, *n* (%)	2 (8.7%)	1 (12.5%)	1 (6.7%)	1.000
Smoking history, *n* (%)	6 (26.1%)	3 (37.5%)	3 (20.0%)	0.621
Alcohol consumption, *n* (%)	5 (21.7%)	3 (37.5%)	2 (13.3%)	0.297
Clinical manifestations				
Chest or back pain, *n* (%)	5 (21.7%)	3 (37.5%)	2 (13.3%)	0.297
Limb weakness, *n* (%)	13 (56.5%)			
Left-sided	11 (47.8%)	3 (37.5%)	8 (53.3%)	0.667
Right-sided	2 (8.7%)	1 (12.5%)	1 (6.7%)	1.000
Altered consciousness, *n* (%)	15 (65.2%)	5 (62.5%)	10 (66.7%)	1.000
Vital signs and laboratory findings				
Admission systolic BP (mmHg, mean ± SD)	115.1 ± 42.7	134.5 ± 49.7	104.8 ± 36.1	0.165
Admission diastolic BP (mmHg, mean ± SD)	65.3 ± 26.7	80.1 ± 31.6	57.5 ± 20.7	0.185
D-dimer (mg/L, median [IQR])	22.73 (9.13–61.17)	7.06 (2.96–23.69)	35.94 (14.83–70.80)	0.012
NIHSS score (median [IQR])	10.0 (6.0–40.0)	9.50 (6.50–33.25)	10.0 (3.0–40.0)	1.000
Diagnosis and treatment				
Intravenous thrombolysis, *n* (%)	9 (39.1%)	1 (12.5%)	8 (53.3%)	0.086

Values are presented as mean ± standard deviation (SD), median (interquartile range, IQR), or number (percentage). BMI, body mass index; BP, blood pressure; NIHSS, National Institutes of Health Stroke Scale.

### 3.2 Clinical manifestations

In patients of TAAD presenting with neurological symptoms, the most frequent symptom was altered consciousness (15 cases, 65.2%), followed by limb weakness (13 cases, 56.5%), of which 11 involved left-sided weakness. Only five patients (21.7%) with TAAD presenting with neurological symptoms accompanied by chest or back pain.

### 3.3 Vital signs and laboratory findings

At admission, the mean systolic blood pressure was 115.1 ± 42.7 mmHg, and the mean diastolic blood pressure was 65.3 ± 26.7 mmHg. Bilateral upper-extremity blood pressure was measured in two patients, one of whom had an inter-arm blood pressure difference of >20 mmHg. D-dimer levels were markedly elevated, ranging from 2.67 to 188.18 mg/L, with a median of 22.73 mg/L (IQR, 9.13–61.17).

### 3.4 Diagnosis and treatment

All patients were initially admitted with a presumed diagnosis of neurological disease. The median National Institutes of Health Stroke Scale score at admission was 10 (IQR, 6–40). Nine patients (39.1%) received intravenous thrombolysis before the definitive diagnosis of aortic dissection. The decision of intravenous thrombolysis was made by the physician based on the Chinese guidelines of acute ischemic stroke.

### 3.5 Comparisons between in-hospital survivors and non-survivors

Comparisons between survivors and the in-hospital non-survivors are presented in Table 1. Exploratory between-group analysis showed that patients in the in-hospital non-survivors had a lower BMI (*p* = 0.049) and higher D-dimer levels (*p* = 0.012) than survivors. No significant differences were observed between the two groups with respect to age, sex, medical history, clinical manifestations, blood pressure, NIHSS score, or intravenous thrombolysis (all *p* > 0.05).

### 3.6 Imaging findings

All patients accepted brain CT scan to exclude a hemorrhagic disease, but none of them accepted MRI scan. During the initial in-hospital evaluation, 5 patients underwent echocardiography, which revealed intimal flap tears or dilatation of the ascending aorta, 1 patient underwent carotid ultrasound, which suggested dissection involving the common carotid artery, and 5 patients underwent thoracic CT scans, which initially indicated possible aortic dissection.

In some patients, CTA was performed as the initial diagnostic modality, whereas in others, preliminary imaging findings suggested aortic dissection, and the diagnosis was ultimately confirmed by CTA in all patients.

### 3.7 Outcomes and follow-up

By the time of discharge, 15 patients (65.2%) had died, while 8 (34.8%) survived. No hemorrhagic complications occurred after intravenous thrombolysis. Among the 8 discharged patients, the median follow-up duration was 39 months (IQR, 14–57.75). Two surgically treated patients remain alive, whereas four of the six non-surgically treated patients died during follow-up. The overall long-term survival rate at the latest follow-up (October 2025) was 17.4% (4/23).

## 4 Discussion

This study is a retrospective single-center case series describing the clinical characteristics, diagnostic course, treatment, and outcomes of patients with TAAD who initially presented with neurological symptoms. Our findings highlight the importance of early recognition of TAAD by neurologists, as this fatal condition may closely mimic acute ischemic stroke at initial presentation.

Several clinical characteristics in our cohort may serve as warning signs that should prompt consideration of TAAD in patients with suspected stroke. Chest or back pain is the most common symptom of TAAD. However, previous studies have reported that approximately 17% of patients with aortic dissection do not experience typical chest pain at initial manifestations (4). In our study, only five of 23 patients (21.7%) reported chest or back pain, suggesting that TAAD may be easily overlooked during acute stroke evaluation when neurological deficits dominate the initial presentation.

The TAAD-associated in-hospital mortality in our cohort was 15/23 (65.2%), which appears to be higher than the overall in-hospital mortality of TAAD reported in previous studies (20.3%) (2), possibly reflecting delayed or inappropriate management. Intravenous thrombolysis or mechanical thrombectomy is the standard therapy for acute ischemic stroke. However, when incorrectly applied to patients with undiagnosed aortic dissection, these interventions may lead to propagation of the dissection, cardiac tamponade, or fatal hemorrhage (7). Among the nine patients in our study who received intravenous thrombolysis before TAAD diagnosis, eight died, resulting in an in-hospital mortality of 88.9%, which was higher than that in patients who did not undergo intravenous thrombolysis (10/14, 71.4%), although the difference did not reach statistical significance (*p* = 0.086).

The neurological manifestations observed in our cohort may have been associated with cerebral hypoperfusion or cerebral malperfusion secondary to TAAD. Prior studies have shown that TAAD complicated by cerebral hypoperfusion is associated with an in-hospital mortality risk of >40%, while patients presenting with altered consciousness exhibit mortality rates of up to 60% (3, 8). These findings suggest that cerebral hypoperfusion may contribute to early mortality in TAAD.

Approximately 6% of patients with TAAD develop ischemic stroke or spinal cord infarction due to extension of the dissection into the carotid or intracranial arteries, or embolization from the false lumen, resulting in neurological deficits such as limb weakness, aphasia, or altered consciousness (9). In our cohort, 13 patients (56.5%) presented with unilateral limb weakness, including 11 with left-sided involvement. Previous studies reported that approximately 57.7% of TAAD cases involve the major arch branches (10), which increases the risk of permanent neurological deficits. Additionally, approximately 71% of infarctions reportedly occur in the right cerebral hemisphere (11). This may partly explain the predominance of left-sided limb weakness observed in our cohort, although this observation requires confirmation in larger studies. In our study, 15 patients (65.2%) exhibited altered consciousness, which may have been multifactorial and related to systemic hypotension, global cerebral hypoperfusion, or directly caused by carotid artery involvement from the aortic dissection. A prior study indicated that in patients with TAAD, global hypoxic brain injury occurs more than three times as frequently as focal ischemic lesions (12).

Aortic dissection is often accompanied by secondary hyperfibrinolysis, resulting in markedly elevated D-dimer levels. In our cohort, D-dimer levels ranged from 2.67 to 188.18 mg/L, with a median of 22.73 mg/L (IQR, 9.13–61.17), which was substantially higher than those observed in ischemic stroke (0.97 mg/L) (13). The reported sensitivity of D-dimer levels for the diagnosis of aortic dissection approaches 96.5% (14), supporting its potential as a diagnostic biomarker for suspected TAAD. Previous studies have shown that higher D-dimer levels are associated with adverse outcomes and increased mortality (15). In our cohort, D-dimer levels were significantly higher in non-survivors than in survivors (35.94 [14.83–70.80] vs. 7.06 [2.96–23.69] mg/L, *p* = 0.012), which is consistent with these findings. In this small cohort, higher D-dimer levels were observed among in-hospital non-survivors associated with poor outcomes. However, this exploratory finding requires validation in larger sample size.

TAAD frequently leads to differential blood pressure between the upper limbs due to flow separation between the true and false lumens. Previous studies have shown that a systolic blood pressure difference >20 mmHg between the arms is significantly associated with TAAD, occurring in approximately 14% of cases (15, 16). However, in acute stroke management, the principle of “time is brain” often leads neurologists to prioritize rapid treatment over comprehensive physical examination, and bilateral arm blood pressure measurement is frequently overlooked. In our cohort, only two patients underwent bilateral blood pressure measurement, and one of them demonstrated a systolic difference of >20 mmHg. These findings support considering bilateral arm blood pressure measurement as a simple bedside assessment when TAAD is clinically suspected.

Previous studies have shown that low systolic blood pressure on admission is independently associated with in-hospital mortality in patients with TAAD. When systolic blood pressure is below 120 mmHg, each 10-mmHg increase is associated with a 33% reduction in in-hospital mortality (17). In our cohort, non-survivors had lower admission systolic blood pressure than survivors (104.8 ± 36.1 vs. 134.5 ± 49.7 mmHg), although the difference did not reach statistical significance, likely because of the limited sample size. These findings suggest that unexplained hypotension, especially in patients presenting with stroke-like symptoms, may be a useful clue for considering TAAD.

Although thoracoabdominal CTA remains the gold standard for diagnosing aortic dissection, current stroke management guidelines do not recommend routine thoracoabdominal CTA screening for all patients with suspected stroke. Notably, five patients in this study exhibited echocardiographic findings suggestive of an intimal flap or ascending aortic dilatation, and one patient underwent carotid ultrasound, which revealed the involvement of the common carotid artery. A previous study reported that echocardiographic detection of an intimal flap has a sensitivity and specificity of 89 and 92%, respectively, and the detection of ascending aortic dilatation has a sensitivity and specificity of 92 and 87% (18). Therefore, in the absence of CTA, attention to such indirect imaging findings may help mitigate missed diagnoses.

Although surgical repair remains the mainstay of treatment for TAAD, management is particularly challenging in patients with concomitant neurological deficits (19–21). Previous studies have reported that the 1-year mortality of non-surgically treated TAAD patients is as high as approximately 83.9% (22). By comparison, surgically treated patients have a substantially better long-term prognosis, with a reported 5-year overall survival of approximately 68.2% (23). Consistent with this, overall survival in our cohort appeared to be higher among surgically treated patients than among those managed conservatively. For patients with TAAD presenting with stroke symptoms, the optimal timing of surgery remains uncertain, and treatment decisions should therefore be individualized (24–26). In our study, four patients underwent surgery more than 48 h after symptom onset, with heterogeneous outcomes, further highlighting the need for an individualized approach to surgical timing.

Certain limitations of this study should be acknowledged. First, this was a retrospective single-center case series, which may limit the generalisability of the findings. Second, the sample size was limited, and multivariable logistic regression was not performed, as any model would have been unstable and prone to overfitting. Third, no control group of patients with true acute ischemic stroke was included. Therefore, the diagnostic performance of the identified warning signs could not be evaluated. Accordingly, our findings should be considered exploratory and require validation in larger multicenter studies. Therefore, the clinical features identified in this study should not be interpreted as independent predictors, but rather as exploratory observations that may help generate hypotheses for future prospective studies.

**Figure 1:**
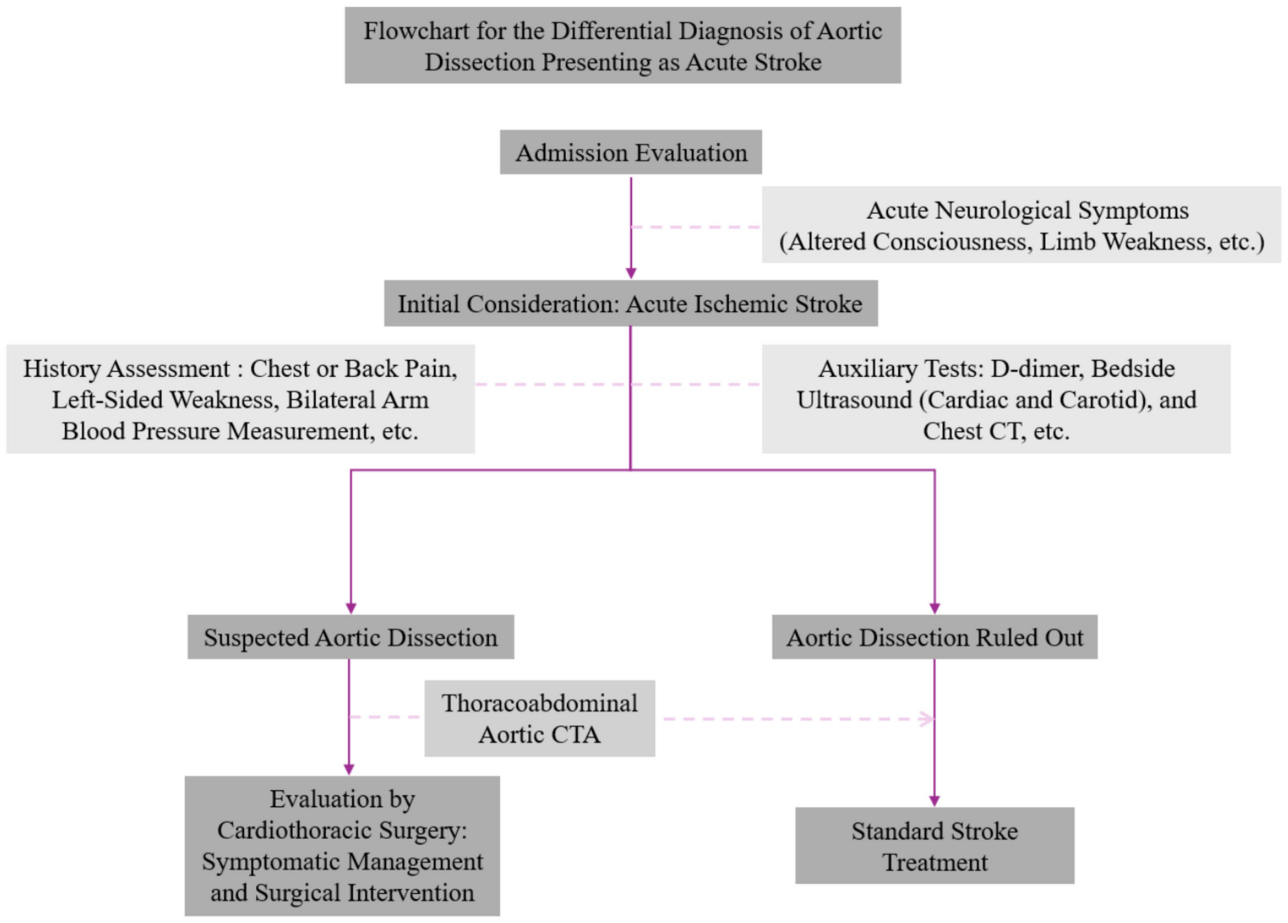
Flowchart for the differential diagnosis of aortic dissection presenting as acute stroke. CT, computed tomography; CTA, computed tomography angiography.

In summary, neurologists should maintain a high index of suspicion for TAAD in patients presenting with suspected stroke, particularly when neurological deficits are accompanied by atypical symptoms, markedly elevated D-dimer levels, or hemodynamic abnormalities. The diagnostic flowchart in Figure 1 highlights key warning signs and decision points that may help neurologists identify potential aortic dissection in patients presenting with stroke-like symptoms. Delayed diagnosis, inappropriate treatment, and delayed surgical intervention may substantially increase the risk of mortality. Early recognition and prompt evaluation are therefore essential to prevent catastrophic outcomes.

## 5 Conclusion

Our findings highlight that acute aortic dissection presenting with stroke–like manifestations remains a significant diagnostic challenge. In patients without typical chest pain, clinical characteristics such as altered consciousness, left-sided weakness, elevated D-dimer levels, and an inter-arm blood pressure difference, when present, may raise suspicion for TAAD. Neurologists should consider bilateral blood pressure assessment and focused vascular imaging in the early evaluation of patients with suspected acute stroke when TAAD is clinically suspected to prevent misdiagnosis and inappropriate thrombolytic therapy. Prompt recognition and timely multidisciplinary management are essential for improving outcomes. Further prospective studies are required to establish the optimal timing of surgery.

## Data Availability

The raw data supporting the conclusions of this article will be made available by the authors upon reasonable request, without undue reservation.
